# Kinematics and temporospatial parameters during gait from inertial motion capture in adults with and without HIV: a validity and reliability study

**DOI:** 10.1186/s12938-020-00802-2

**Published:** 2020-07-24

**Authors:** Karina Berner, John Cockcroft, Quinette Louw

**Affiliations:** 1 Division of Physiotherapy, Faculty of Medicine and Health Sciences, Stellenbosch University, PO Box 241, Cape Town, 8000 South Africa; 2 Central Analytical Facilities, Neuromechanics Unit, Stellenbosch University, Private Bag X1, Matieland, 7602 South Africa

**Keywords:** Gait analysis, Kinematics, Temporospatial parameters, Inertial motion capture, Inertial measurement units, Reliability, Validity, Measurement error, HIV infection

## Abstract

**Background:**

Inertial measurement unit (IMU)-based motion capture systems are gaining popularity for gait analysis outside laboratories. It is important to determine the performance of such systems in specific patient populations. We aimed to validate and determine within-day reliability of an IMU system for measuring lower limb gait kinematics and temporal–spatial parameters (TSP) in people with and without HIV.

**Methods:**

Gait was recorded in eight adults with HIV (PLHIV) and eight HIV-seronegative participants (SNP), using IMUs and optical motion capture (OMC) simultaneously. Participants performed six gait trials. Fifteen TSP and 28 kinematic angles were extracted. Intraclass correlations (ICC), root-mean-square error (RMSE), mean absolute percentage error and Bland–Altman analyses were used to assess concurrent validity of the IMU system (relative to OMC) separately in PLHIV and SNP. IMU reliability was assessed during within-session retest of trials. ICCs were used to assess relative reliability. Standard error of measurement (SEM) and percentage SEM were used to assess absolute reliability.

**Results:**

Between-system TSP differences demonstrated acceptable-to-excellent ICCs (0.71–0.99), except for double support time and temporophasic parameters (< 0.60). All TSP demonstrated good mean absolute percentage errors (≤7.40%). For kinematics, ICCs were acceptable to excellent (0.75–1.00) for all but three range of motion (ROM) and four discrete angles. RMSE and bias were 0.0°–4.7° for all but two ROM and 10 discrete angles. In both groups, TSP reliability was acceptable to excellent for relative (ICC 0.75–0.99) (except for one temporal and two temporophasic parameters) and absolute (%SEM 1.58–15.23) values. Reliability trends of IMU-measured kinematics were similar between groups and demonstrated acceptable-to-excellent relative reliability (ICC 0.76–0.99) and clinically acceptable absolute reliability (SEM 0.7°–4.4°) for all but two and three discrete angles, respectively. Both systems demonstrated similar magnitude and directional trends for differences when comparing the gait of PLHIV with that of SNP.

**Conclusions:**

IMU-based gait analysis is valid and reliable when applied in PLHIV; demonstrating a sufficiently low precision error to be used for clinical interpretation (< 5° for most kinematics; < 20% for TSP). IMU-based gait analysis is sensitive to subtle gait deviations that may occur in PLHIV.

## Background

Ever since the first definition of HIV/AIDS in the 1980s, motor impairments were noted and described as defining characteristics of the disease [1]. Unfortunately, motor function remains compromised in people living with HIV (PLHIV) even in the current era of modern antiretroviral therapy (ART) [2]. The HI-virus itself, ART drug toxicity, interactions between various comorbidities, traditional risk factors and synergistic mechanisms to usual aging may all contribute to the observed impairments. Common impairments include muscle weakness and dynapenia [3], peripheral sensory neuropathies [4], motor slowing and postural imbalance associated with white matter alterations [5]. Furthermore, a state of ongoing inflammation or immune activation may cause PLHIV to experience non-AIDS-defining complications resembling geriatric processes (including falls and fractures) at relatively young ages [6].

Indeed, about one-third of young- to middle-aged PLHIV experience falls [7, 8] and relatively young PLHIV seem to have walking impairments resembling fall-predisposing gait characteristics in older adults [9]. However, the true gait pattern in PLHIV remains inconclusive, as the gait characteristics that have been assessed are limited to gait speed, cadence and gait initiation time (slowed gait speed being the most consistent observation, while delayed fast gait initiation time and low cadence have been described in PLHIV who are also obese) [9]. Furthermore, these observations remain limited to semi-quantitative clinical assessments, meaning that subtle and early impairments in a young population may remain undocumented.

There is currently no evidence from three-dimensional (3D) gait analysis describing the gait patterns of PLHIV. This situation prohibits a sensitive evaluation of movement quality and level of impairment and therefore little understanding of the impact of potential impairments remains. Despite the many parallels that have been drawn between usual aging and the processes associated with HIV disease or treatment (e.g., telomere shortening, increased interleukin-6, reduced bone mineral density), it must be noted that the etiological patterns of chronological aging likely differ from accelerated or accentuated aging due to HIV and/or ART. Different patterns of impairment or functional decline may manifest in younger adults dealing with complex chronic illnesses (such as HIV) and the associated treatment burden compared to the general population of older adults [10]. As the effective, targeted rehabilitation of gait function largely depends on an understanding of the underlying impairments and their interactions, there is a need to more rigorously investigate the gait patterns that may be unique to PLHIV.

Instrumented motion analysis provides 3D data that are accurate and precise. Such quantitative data can more comprehensively describe gait patterns and (even subtle) impairments; supporting clinical decision-making and allowing for early diagnosis and intervention [11]. Although marker-based optical motion capture (OMC) remains the gold standard for human motion capture, inertial motion capture systems are increasingly used for 3D gait analysis in various settings outside of the gait laboratory. Inertial motion capture offers several pragmatic benefits relative to OMC. Such systems are more compact, affordable, portable and user-friendly; making them ideal for use in clinical environments [12]. Inertial motion capture is based on small yet powerful integrated circuits (inertial measurement units or IMUs); typically comprising on-board tri-axial gyroscopes, tri-axial magnetometers and tri-axial accelerometers. Using sensor fusion techniques, the ability of IMUs to accurately track orientation has become advanced [13].

However, IMU output is body-referenced (i.e., absolute skeletal position is not readily available to IMUs), suffers from drifts and ferromagnetic disturbances that need correction [14], and often uses automated processing of measured data to generate time and space parameters (TSP). The user may not be able to interfere in such processing. It is thus important to determine the validity of automatically calculated gait events such as initial contact and toe-off, which are important for determining gait phases and other TSP and for understanding joint motion at specific points and phases of the gait cycle [15].

Although the validity and reliability of IMUs have been investigated in healthy participants and certain patient groups, underlying assumptions and body models may not be the same for other population groups or pathologies [16]. IMU validity and reliability should thus be demonstrated in the condition of intended use [16] to improve the quality of data collection and interpretation. Since the 3D gait patterns of PLHIV have never been reported, it remains unknown which biomechanical impairments they might demonstrate and validation in this population is therefore warranted. In addition, although a recent review [17] reported that IMUs are valid for assessing whole body range of motion (ROM), evidence for reliability is lacking and there is a paucity of studies reporting on comprehensive, clinically relevant gait outcomes [18]. This study therefore aimed to determine the concurrent validity of an IMU system (versus OMC and the Conventional Gait Model as reference standard), and the within-session, between-trial reliability of IMUs for measuring lower limb kinematic and temporospatial gait outcomes in PLHIV and HIV-seronegative participants (SNP). The study further aimed to determine whether a gait analysis conducted using IMUs would differentiate between gait outcomes of PLHIV and SNP in a similar manner to a gait analysis conducted using OMC.

## Results

The full set of data for all participants (*n* = 8 PLHIV and *n* = 8 SNP) were analyzed for both the IMU and OMC systems (a total of 96 gait trials for each system). IMU-detected gait events (initial contact and toe-off) demonstrated differences from OMC-detected events as follows: initial contact errors for IMUs demonstrated median (interquartile range [IQR]) values of − 10.00 ms (96.25 ms) in SNP and − 7.50 ms (82.50 ms) in PLHIV. Median (IQR) toe-off errors for IMUs were 2.50 ms (95.00 ms) in SNP and − 5.00 ms (62.5 ms) in PLHIV.

### Concurrent validity: temporal, spatial, temporophasic and temporospatial parameters (TSP)

**Fig. 1:**
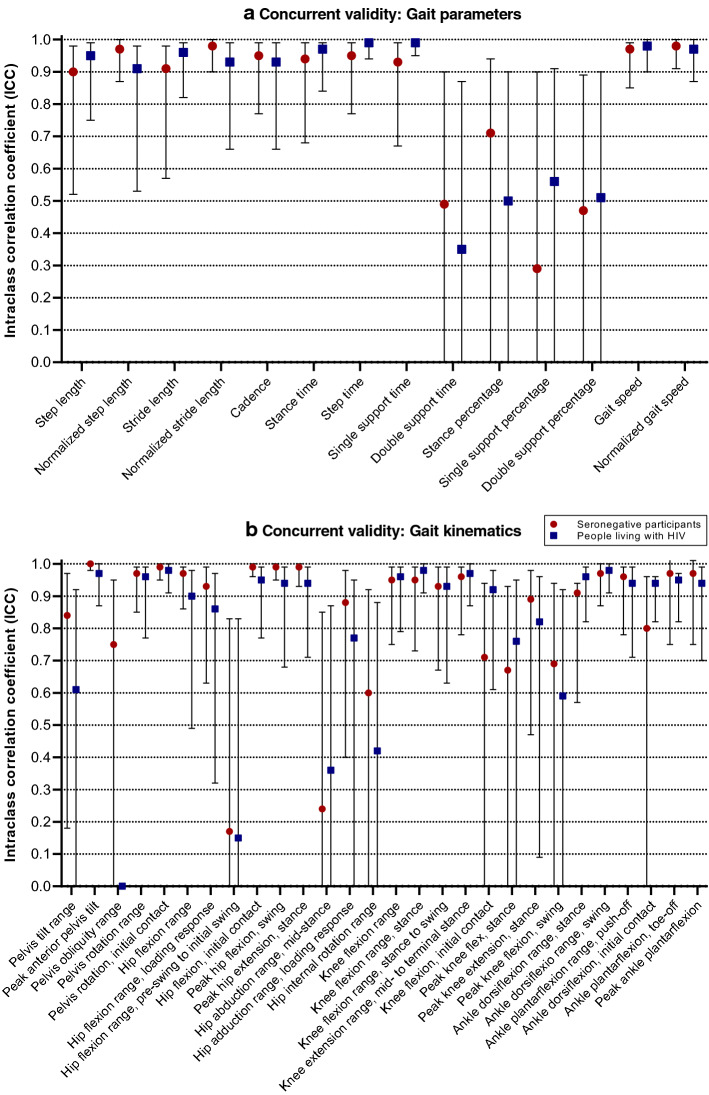
Validity of gait parameters and kinematics measured by inertial measurement units (relative to optical motion capture). Error bars indicate 95% confidence intervals

Between-system differences for TSP demonstrated acceptable-to-excellent intraclass correlation coefficients (ICCs, 0.71–0.99), except for double support time and temporophasic parameters, which demonstrated questionable to poor ICCs (< 0.60) (Fig. 1). All TSP demonstrated good mean absolute percentage errors (≤ 7.40%). RMSE, bias and limits of agreement (LoA) between IMUs and OMC were close to zero for temporal, leg length-normalized spatial and temporospatial parameters in both participant groups. Mean absolute percentage errors were < 2.68% for all parameters except double support time (7.40% and 5.79% for SNP and PLHIV, respectively) and double support percentage (6.96% and 5.69% for SNP and PLHIV, respectively). These latter parameters had the largest mean absolute percentage errors in both groups (Table 1).

**Table 1:** Concurrent validity of gait parameters measured by inertial measurement units (relative to optical motion capture)

Parameter	RMSE, mean (SD)	MAPE, %	Bias	LoA	RMSE, mean (SD)	MAPE, %	Bias	LoA
	SNP				PLHIV			
Spatial parameters								
Step length (cm)	3.22 (1.63)	1.66	− 1.05	− 5.83; 3.72	4.38 (2.01)	1.07	− 0.74	− 4.95; 3.47
Normalized step length	0.04 (0.02)	1.66	− 0.01	− 0.07; 0.04	0.05 (0.02)	1.07	− 0.01	− 0.05; 0.04
Stride length	4.46 (3.51)	2.51	− 3.26	− 11.45; 4.92	4.46 (2.62)	2.28	− 2.95	− 10.68; 4.78
Normalized stride length	0.05 (0.04)	2.51	− 0.04	− 0.13; 0.05	0.05 (0.03)	2.28	− 0.03	− 0.11; 0.05
Temporal parameters								
Cadence (steps/min)	6.93 (4.41)	0.39	0.49	− 5.55; 6.54	6.15 (4.86)	0.18	0.01	− 4.75; 5.00
Stance time (s)	0.02 (0.01)	1.22	0.01	− 0.03; 0.04	0.03 (0.02)	1.06	0.01	− 0.04; 0.05
Step time (s)	0.03 (0.02)	0.12	< 0.00	− 0.03; 0.03	0.03 (0.02)	0.17	< 0.00	− 0.02; 0.02
Single support time (s)	0.02 (0.01)	1.21	− 0.01	− 0.03; 0.02	0.02 (0.01)	1.43	− 0.01	− 0.02; 0.01
Double support time (s)	0.03 (0.01)	7.40	0.01	− 0.04; 0.06	0.03 (0.03)	5.79	0.01	− 0.05; 0.07
Temporophasic parameters								
Stance percentage (%GC)	1.72 (0.39)	0.98	0.57	− 1.37; 2.51	1.97 (1.23)	1.13	0.69	− 2.00; 3.43
Single support percentage (%GC)	2.44 (1.35)	1.37	− 0.57	− 3.53; 2.39	2.31 (1.40)	1.26	− 0.50	− 3.24; 2.24
Double support percentage (%GC)	3.01 (1.14)	6.96	1.14	− 3.58; 5.86	2.68 (2.29)	5.69	1.18	− 4.14; 6.51
Temporospatial parameters								
Gait speed (m/s)	0.04 (0.03)	2.68	− 0.03	− 0.11; 0.04	0.04 (0.03)	2.13	− 0.03	− 0.10; 0.05
Normalized gait speed	0.01 (0.01)	2.68	− 0.01	− 0.04; 0.01	0.01 (0.01)	2.13	− 0.01	− 0.03; 0.02

*MAPE* mean absolute percentage error, *GC* gait cycle, *LoA* limits of agreement, *PLHIV* people living with HIV-1 infection, *RMSE* root-mean-square error, *SNP* HIV-seronegative participants

### Concurrent validity: kinematics

For kinematics, ICCs were acceptable to excellent (0.75–1.00) for all but three ROM and four discrete angles (Fig. 1). RMSE and bias were 0.0°–4.7° for all but two ROM and 10 discrete angles. RMSE, biases and LoA were generally larger in PLHIV (although remaining within 2° from those observed in SNP). Between-system differences were < 5° for all ROM outcomes; except for hip internal rotation (both groups) and hip flexion [in PLHIV: ROM over entire gait cycle (i.e., between two successive occurrences of ipsilateral initial contact) and ROM from pre-swing to initial swing (i.e., from contralateral initial contact to the instant when the ipsilateral swing leg is adjacent to the stance limb)], while angular values at specific time points of the gait cycle tended to exceed 5° (Table 2).

**Table 2:** Concurrent validity of kinematic angles measured by inertial measurement units (relative to optical motion capture)

Angle/ROM (degrees)	RMSE, mean (SD)	Bias	LoA	RMSE, mean (SD)	Bias	LoA
	SNP			PLHIV		
Pelvis						
Pelvis tilt ROM	2.1 (1.1)	2.0	− 0.1; 4.1	2.9 (1.8)	2.2	− 2.2; 6.5
Peak anterior pelvis tilt	10.2 (7.1)	− 10.1	− 24.4; 4.2	12.5 (5.9)	− 12.5	− 24.2; − 0.8
Pelvis obliquity ROM	2.4 (1.7)	− 0.6	− 6.2; 5.0	4.7 (3.3)	1.7	− 9.3; 12.7
Pelvis rotation ROM	3.3 (1.7)	3.0	− 0.6; 6.6	4.1 (2.4)	3.7	− 1.2; 8.6
Pelvis rotation, IC	5.5 (2.0)	1.2	− 1.0; 3.5	5.7 (4.5)	2.2	− 1.0; 5.5
Hip						
Hip flexion ROM	3.5 (1.1)	− 3.1	− 6.0; − 0.2	5.5 (2.1)	− 5.0	− 9.1; − 1.0
Hip flexion ROM, LR	1.9 (1.5)	0.5	− 1.3; 2.3	2.3 (1.3)	1.3	− 2.0; 4.6
Hip flexion ROM, PS to IS (H3)	3.1 (1.4)	< 0.0	− 5.2; 5.1	5.1 (4.4)	− 1.6	− 10.4; 7.2
Hip flexion, IC	8.7 (5.1)	− 5.4	− 22.0; 11.3	10.6 (4.5)	− 9.4	− 22.1; 3.4
Peak hip flexion, swing	7.8 (4.8)	− 3.7	− 20.1; 12.7	10.0 (4.4)	− 9.0	− 21.0; 3.0
Peak hip extension, stance	7.3 (3.9)	0.6	− 16.0; 17.1	6.7 (3.7)	4.0	− 8.8; 16.7
Hip abduction ROM, MSt	3.2 (1.7)	< 0.0	− 5.3; 5.4	4.5 (3.1)	3.3	− 3.1; 9.6
Hip adduction ROM, LR	3.2 (1.4)	2.8	− 0.1; 5.6	2.9 (1.9)	2.5	− 1.4; 6.3
Hip internal rotation ROM	5.2 (1.4)	1.7	− 6.0; 9.5	5.7 (3.3)	2.7	− 6.7; 12.1
Knee						
Knee flexion ROM	2.2 (0.9)	0.4	− 3.1; 3.9	3.0 (1.5)	1.4	− 3.3; 6.0
Knee flexion ROM, stance (K1)	2.8 (1.3)	1.6	− 1.9; 5.1	1.9 (1.7)	0.7	− 2.0; 3.4
Knee flexion ROM, stance to swing (K3)	2.5 (1.3)	− 1.7	− 5.1; 1.6	2.6 (1.3)	− 1.5	− 5.5; 2.5
Knee extension ROM, MSt to TSt (K2)	2.5 (1.3)	− 2.1	− 4.9; 0.7	3.4 (1.8)	− 3.0	− 6.7; 0.6
Knee flexion, IC	7.8 (1.9)	5.8	− 2.2; 13.7	4.8 (3.3)	2.2	− 6.3; 10.7
Peak knee flexion, stance	15.3 (7.5)	14.2	− 0.4; 28.8	11.0 (7.5)	9.8	− 5.5; 25.0
Peak knee extension, stance	9.3 (2.8)	− 8.3	− 1.1; − 15.5	5.3 (4.0)	− 4.0	− 12.5; 4.6
Peak knee flexion, swing	8.5 (2.5)	7.6	1.2; 14.1	5.4 (3.6)	4.1	− 4.1; 12.3
Ankle						
Ankle dorsiflexion ROM, stance (A1)	2.7 (1.1)	2.0	− 1,1; 5.2	2.7 (0.7)	1.7	− 1.5; 4.9
Ankle dorsiflexion ROM, swing	2.3 (1.8)	1.1	− 3.3; 5.4	2.4 (1.0)	1.1	− 2.3; 4.6
Ankle plantarflexion ROM, push off (A2)	2.6 (1.7)	0.5	− 4.8; 5.8	3.4 (1.6)	2.2	− 2.7; 7.1
Ankle dorsiflexion, IC	3.6 (1.7)	− 2.8	− 7.3; 1.7	5.5 (1.8)	− 4.6	− 10.8; 1.6
Ankle plantarflexion, TO	4.1 (1.3)	1.9	− 4.1; 7.9	6.1 (3.3)	5.1	− 1.7; 11.8
Peak ankle plantarflexion	4.3 (2.1)	1.1	− 6.9; 9.2	5.4 (2.9)	4.6	− 2.7; 11.9

*A1* corresponding to A1 power phase of ankle, *A2* corresponding to A2 power phase of ankle, *CI* confidence interval, *H3* corresponding to H3 power phase of hip, *HR* heel rise, *IC* initial contact, *ICC* intraclass correlation coefficient, *K1* corresponding to K1 power phase of knee, *K2* corresponding to K2 power phase of knee, *K3* corresponding to K3 power phase of knee, *LoA* limits of agreement, *LR* loading response, *MSt* mid-stance, *PLHIV* people living with HIV-1-infection, *RMSE* root-mean-square error, *SNP* HIV-seronegative participants

### Within-session, between-trial reliability: TSP

**Fig. 2:**
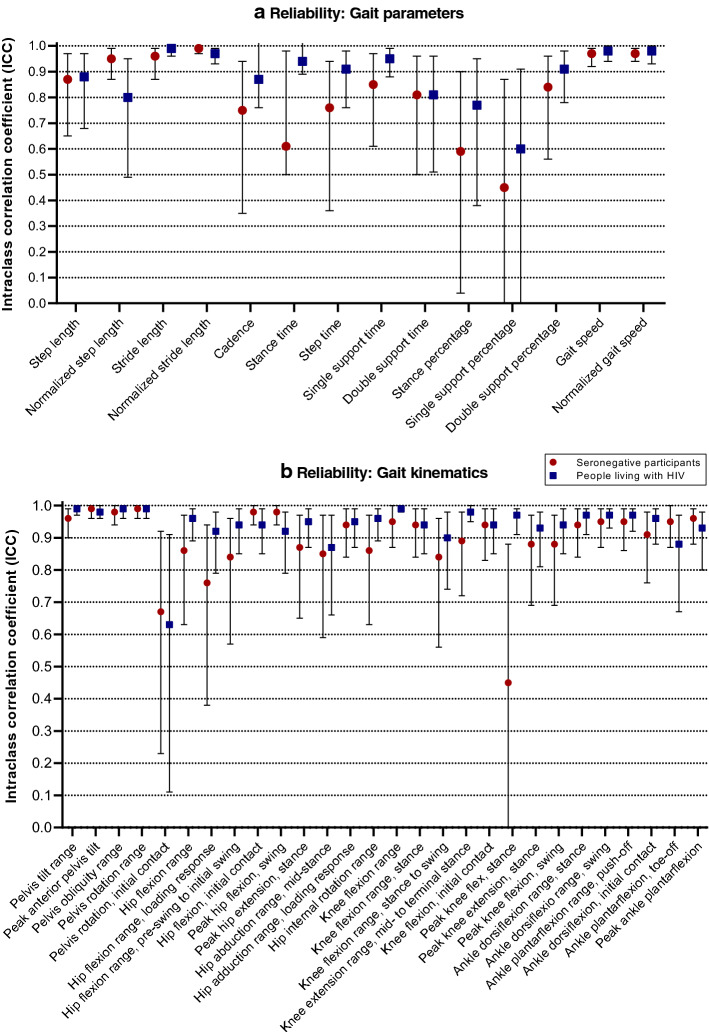
Relative reliability of gait parameters and kinematics measured by inertial measurement units. Error bars indicate 95% confidence intervals

In both participant groups, TSP reliability was acceptable to excellent for relative values (ICC 0.75–0.99) (except for stance time and percentage in SNP and single support percentage in both groups) (Fig. 2) as well as for absolute values [percentage standard error of measurement (%SEM) 1.58–15.23]. Spatial parameters showed better absolute reliability in SNP [lower standard error of measurement (SEM), %SEM and upper 95% confidence limit (CL)], temporal and temporophasic parameters were more reliable in PLHIV and temporospatial parameters were more reliable in SNP. However, for all these outcomes,  %SEM observed in the two participant groups were within ~ 2% of each other (Table 3).

**Table 3:** Absolute reliability (measurement error) of gait parameters measured by inertial measurement units

Parameter	SEM	%SEM	Upper 95% CL	SEM	%SEM	Upper 95% CL
	SNP			PLHIV		
Spatial parameters						
Step length (cm)	4.01	6.09	4.81	4.33	6.82	5.20
Normalized step length	0.04	5.20	0.05	0.05	6.98	0.06
Stride length (cm)	3.20	2.46	3.84	4.03	3.23	4.84
Normalized stride length	0.04	2.65	0.05	0.05	3.56	0.06
Temporal parameters						
Cadence (steps/min)	8.31	7.08	9.97	9.67	8.82	11.60
Normalized cadence	2.45	7.04	2.94	2.83	8.57	3.40
Stance time (s)	0.06	9.78	0.07	0.03	4.48	0.04
Step time (s)	0.04	7.70	0.05	0.04	7.22	0.05
Single support time (s)	0.03	7.14	0.04	0.03	6.91	0.04
Double support time (s)	0.03	15.23	0.04	0.03	12.77	0.04
Temporophasic parameters						
Stance percentage (%GC)	1.97	3.31	2.36	1.60	2.63	1.92
Single support percentage (%GC)	2.56	6.34	3.07	2.49	6.32	2.99
Double support percentage (%GC)	2.18	11.42	2.62	2.30	10.71	2.76
Temporospatial parameters						
Gait speed (m/s)	0.02	1.58	0.02	0.02	1.75	0.02
Normalized gait speed	0.04	9.20	0.05	0.04	10.34	0.05

SEM 95% CL were calculated using a sample-and-trial-specific multiplying factor of 1.2 [19]

*%SEM* absolute percentage SEM, *CL* confidence limits of SEM, *GC* gait cycle, *PLHIV* people living with HIV-1 infection, *SEM* standard error of measurement, *SNP* HIV-seronegative participants

### Within-session, between-trial reliability: kinematic angles

The reliability of IMU-measured kinematic angles was similar between participant groups and demonstrated acceptable-to-excellent relative reliability (ICC 0.76–0.99, except for pelvis rotation at initial contact and peak knee flexion during stance) (Fig. 2). Clinically acceptable absolute reliability was demonstrated (SEM 0.7°–4.4°) for all but three discrete angles (Table 4). In SNP, pelvis rotation at initial contact and ankle plantarflexion angle at toe-off were the only angles with absolute reliability exceeding 5°, while in PLHIV, peak knee flexion in stance showed an SEM of 5.8°, with an upper 95% CL of 7.0°.

**Table 4:** Absolute reliability (measurement error) of kinematic angles measured by inertial measurement units

Angle/ROM (degrees)	SNP		PLHIV	
	SEM	Upper 95% CL	SEM	Upper 95% CL
Pelvis				
Pelvis tilt ROM	0.7	0.8	0.8	1.0
Peak pelvis anterior tilt	1.1	1.3	1.2	1.4
Pelvis obliquity ROM	0.9	1.1	1.1	1.3
Pelvis rotation ROM	2.1	2.5	1.8	2.2
Pelvis rotation, IC	5.2	6.2	3.7	4.4
Hip				
Hip flexion ROM	2.4	2.9	2.1	2.5
Hip flexion ROM, LR	1.7	2.0	2.6	3.1
Hip flexion ROM, PS to IS (H3)	2.3	2.8	2.0	2.4
Hip flexion, IC	2.4	2.9	2.1	2.5
Peak hip flexion, swing	2.2	2.6	2.1	2.5
Peak hip extension, stance	1.8	2.2	2.4	2.9
Hip abduction ROM, mid-stance	2.6	3.1	2.0	2.4
Hip adduction ROM, LR	1.4	1.7	1.5	1.8
Hip internal rotation ROM	2.6	3.1	2.8	3.4
Knee				
Knee flexion ROM	1.4	1.7	2.2	2.6
Knee flexion ROM, stance (K1)	2.9	3.5	2.5	3.0
Knee flexion ROM, stance to swing (K3)	3.0	3.6	2.8	3.4
Knee extension ROM, MSt to TSt (K2)	1.9	2.3	2.4	2.9
Knee flexion, IC	3.0	3.6	3.2	3.8
Peak knee flexion during stance, LR	3.9	4.7	5.8	7.0
Peak knee extension, stance	2.9	3.5	3.1	3.7
Peak knee flexion, swing	2.0	2.4	2.9	3.5
Ankle				
Ankle dorsiflexion ROM, stance (A1)	2.0	2.4	1.9	2.3
Ankle dorsiflexion ROM, swing	2.9	3.5	3.3	4.0
Ankle plantarflexion ROM, push off (A2)	2.2	2.6	3.7	4.4
Ankle dorsiflexion, IC	2.0	2.4	2.1	2.5
Ankle plantarflexion, TO	5.2	6.2	4.4	5.3
Peak ankle plantarflexion	4.1	4.9	4.4	5.3

*A1* corresponding to A1 power phase of ankle, *A2* corresponding to A2 power phase of ankle, *H3* corresponding to H3 power phase of hip, *HR* heel rise, *IC* initial contact, *K1* corresponding to K1 power phase of knee, *K2* corresponding to K2 power phase of knee, *K3* corresponding to K3 power phase of knee, *LR* loading response, *MSt* mid-stance, *PLHIV* people living with HIV-1 infection, *ROM* range of motion, *SEM* standard error of measurement, *SNP* HIV-seronegative participants, *TO* toe-off, *TSt* terminal stance

### Between-group comparisons (performed separately for IMU and OMC)

**Fig. 3:**
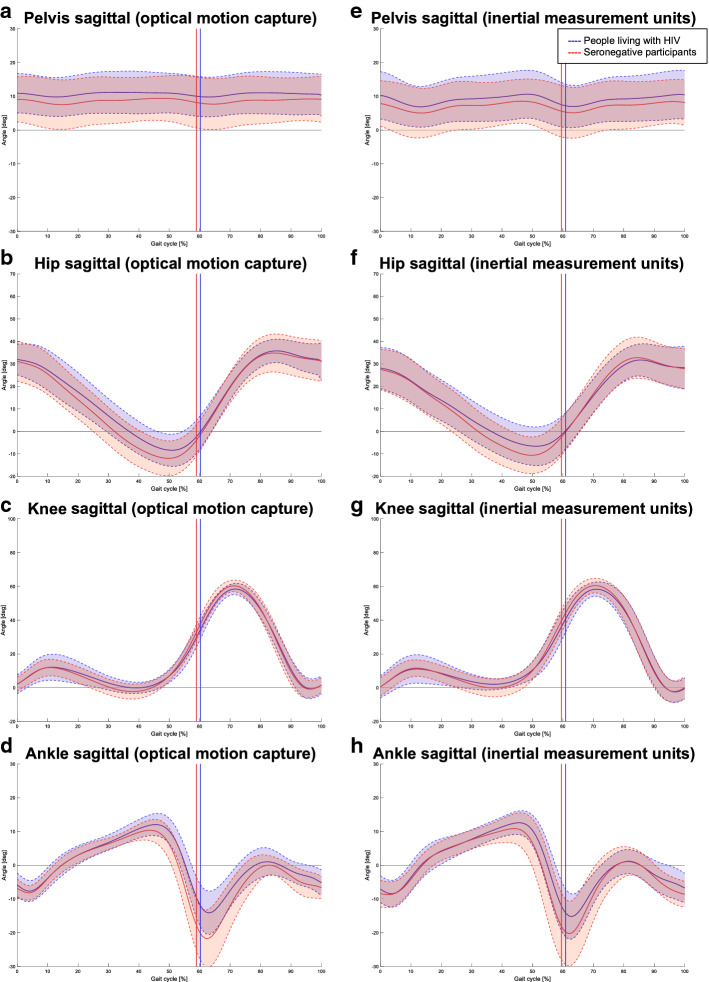
Comparative gait traces for people living with HIV (PLHIV) and HIV-seronegative participants (SNP). Sagittal plane kinematic traces are shown as measured by optical motion capture (OMC) (**a**–**d**) and inertial measurement units (IMUs) (**e**–**h**). The graphs illustrate the mean (solid lines) ± standard deviation (shaded areas bounded by dashed lines) estimated by each system for each group. Note similarities in magnitude and direction of differences between PLHIV and SNP as observed by each system

Between-group differences are presented here to demonstrate the validity of a clinical gait assessment by both instrumented systems. Selected kinematic gait curves for both groups and systems are presented in Fig. 3 (only sagittal plane traces shown). Directional trends of between-group differences were largely similar in IMU and OMC results for TSP (Table 5) and kinematics angles (Table 6). For TSP, between-group differences and p-values were almost identical for both systems, but less so for temporophasic outcomes. In PLHIV, both OMC and IMUs demonstrated significantly increased stance- and double-support times, as well as a clinically (but not statistically) significantly slowed gait speed (> 0.1 m/s). For kinematics, in terms of clinical significance (differences > 5°), both systems demonstrated reduced values in PLHIV for ankle dorsiflexion ROM during swing and peak ankle plantarflexion.

**Table 5:** Gait parameter differences between people with and without HIV as measured by the respective systems

Parameter	Mean difference	95% CI	*p*-value	Mean difference	95% CI	*p*-value
	IMU			OMC		
Spatial parameters						
Step length (cm)	2.29	− 2.92; 7.50	0.36	2.60	− 1.9; 7.1	0.24
Normalized step length	0.05	− 0.03; 0.13	0.19	0.10	0.0; 0.1	0.09
Stride length (cm)	5.31	− 5.11; 15.73	0.29	5.62	− 3.52; 14.76	0.21
Normalized stride length	0.11	− 0.04; 0.26	0.15	0.11	− 0.01; 0.24	0.08
Temporal parameters						
Cadence (steps/min)	7.71	− 2.11; 17.54	0.11	7.34	− 2.41; 17.09	0.13
Stance time (s)	− 0.06	− 0.11; 0.00	0.04*	− 0.06	− 0.11; 0.00	0.04*
Step time (s)	− 0.04	− 0.09; 0.01	0.12	− 0.04	− 0.09; 0.01	0.11
Single support time (s)	− 0.02	− 0.06; 0.03	0.41	− 0.02	− 0.06; 0.02	0.37
Double support time (s)	− 0.04	− 0.07; − 0.01	0.01*	− 0.04	− 0.06; − 0.02	< 0.01*
Temporophasic parameters						
Stance time (%GC)	− 1.41	− 2.90; 0.09	0.06	− 1.29	− 2.13; − 0.45	0.01*
Single support time (%GC)	0.99	− 0.65; 2.62	0.22	1.06	0.20; 1.92	0.02*
Double support time (%GC)	− 2.39	− 5.34; 0.56	0.10	− 2.35	− 3.97; − 0.73	0.01*
Temporospatial parameters						
Gait speed (m/s)	0.12	− 0.02; 0.26	0.09	0.13	0.00; 0.27	0.06
Normalized gait speed	0.05	0.00; 0.10	0.06	0.05	0.00; 0.10	0.03*

Asterisks indicate statistically significant differences between participant groups

*%GC* percentage of gait cycle, *IMU* inertial measurement units, *OMC* optical motion capture

**Table 6:** Kinematic differences between people with and without HIV as measured by the respective systems

Kinematic angle	Mean difference	95% CI	*p*-value	Mean difference	95% CI	*p*-value
	IMU			OMC		
Ankle						
Ankle dorsiflexion ROM during stance (A1)	− 0.3°	− 4.1°; 3.5°	0.87	− 0.6°	− 4.7°; 3.5°	0.76
Ankle dorsiflexion ROM during swing	6.4°*	− 1.2°; 13.9°	0.09	6.4°*	− 0.6°; 13.4°	0.07
Ankle plantarflexion ROM during push off (A2)	4.3°	− 2.3°; 11.0°	0.18	6.1°*	− 0.3°; 12.4°	0.06
Ankle dorsiflexion angle at IC	− 1.5°	− 4.8°; 1.9°	0.36	− 1.2°	− 4.2°; 1.8°	0.42
Ankle plantarflexion angle at TO	4.6°	− 2.1°; 11.4°	0.16	5.6°*	0.0°; 11.3°	0.05
Peak ankle plantarflexion during GC	5.9°*	− 1.6°; 13.4°	0.11	7.2°*	0.2°; 14.3°	0.05
Knee						
Knee flexion ROM during GC	2.6°	− 3.3°; 8.4°	0.36	3.6°	− 1.4°; 8.6°	0.15
Knee flexion ROM during stance (K1)	0.3°	− 4.7°; 5.4°	0.90	− 0.6°	− 5.4°; 4.3°	0.80
Knee flexion ROM from stance to swing (K3)	3.9°	0.0°; 7.8°	0.05	4.1°	0.2°; 8.1°	0.04*
Knee extension ROM, MSt to TSt (K2)	2.7°	− 2.3°; 7.8°	0.26	1.8°	− 3.5°; 7.1°	0.47
Knee flexion at IC	0.0°	− 5.3°; 5.3°	1.00	0.1°	− 4.1°; 4.4°	0.95
Peak knee flexion during stance, LR	− 1.1°	− 6.8°; 4.6°	0.69	− 1.8°	− 7.3°; 3.7°	0.49
Peak knee extension during stance	1.0°	− 3.4°; 5.5°	0.63	1.7°	− 1.6°; 5.0°	0.30
Peak knee flexion during swing	1.9°	− 0.8°; 4.6°	0.16	2.0°	− 0.5°; 4.6°	0.11
Hip						
Hip flexion ROM during GC	4.6°	0.2°; 9.0°	0.04*	2.6°	− 1.5°; 6.7°	0.19
Hip flexion ROM during LR	− 1.2°	− 3.5°; 1.1°	0.28	− 0.4°	− 2.7°; 1.8°	0.69
Hip flexion ROM, PS to IS (H3)	0.4°	− 3.1°; 3.8°	0.83	− 1.2°	− 3.4°; 1.1°	0.28
Hip flexion angle at IC	− 0.66°	− 10.4°; 9.3°	0.90	− 0.8°	− 9.3°; 7.8°	0.85
Peak hip flexion during swing	0.7°	− 8.4°; 9.7°	0.87	− 0.8°	− 8.4°; 6.9°	0.84
Peak hip extension during stance	3.7°	− 5.2°; 12.7°	0.39	3.3°	− 4.8°; 11.5°	0.397
Hip abduction ROM during mid-stance	− 0.4°	− 3.4°; 2.6°	0.79	2.8°	0.9°; 4.8°	0.01*
Hip adduction ROM during loading response	1.6°	− 1.2°; 4.4°	0.24	1.3°	− 0.7°; 3.2°	0.18
Hip internal rotation ROM during GC	1.1°	− 3.1°; 5.3°	0.59	2.1°	− 1.9°; 6.0°	0.28
Pelvis						
Pelvis tilt ROM during GC	0.5°	− 1.9°; 3.0°	0.65	0.7°	− 0.4°; 1.9°	0.20
Peak pelvis anterior tilt during GC	− 1.6°	− 9.6°; 6.3°	0.66	− 1.4°	− 8.6°; 5.8°	0.69
Pelvis obliquity ROM during GC	1.2°	− 2.0°; 4.4°	0.44	3.5°	− 0.4°; 7.5°	0.08
Pelvis rotation ROM during GC	0.6°	− 6.2°; 7.4°	0.85	1.4°	− 4.0°; 6.8°	0.60
Pelvis rotation at IC	0.5°	− 2.8°; 3.9°	0.74	0.7°	− 2.9°; 4.3°	0.68

Asterisks indicate clinically and/or statistically significant differences between participant groups

*A1* corresponding to A1 power phase of ankle, *A2* corresponding to A2 power phase of ankle, *H3* corresponding to H3 power phase of hip, *HR* heel rise, *IC* initial contact, *K1* corresponding to K1 power phase of knee, *K2* corresponding to K2 power phase of knee, *K3* corresponding to K3 power phase of knee, *LR* loading response, *MSt* mid-stance, *PLHIV* people living with HIV-1 infection, *ROM* range of motion, *SEM* standard error of measurement, *SNP* HIV-seronegative participants, *TO* toe-off, *TSt* terminal stance

## Discussion

This study assessed the validity and reliability of 3D gait analyses in PLHIV and community-matched SNP using a body-worn IMU system relative to a camera-based OMC system. PLHIV are suggested to suffer subtle gait impairments that may predispose them to adverse functional outcomes. This is the first study to suggest the use of IMUs in PLHIV for measuring a comprehensive set of clinically relevant gait outcomes as a reliable alternative to OMC. In both participant groups, and for most outcomes, the validity (estimated by between-system comparison) and reliability (estimated from repeated testing) of IMU-measured TSP and lower limb angles were deemed acceptable for detecting clinically meaningful differences [20]. In terms of absolute reliability, i.e., measurement error, all 43 gait analysis outcomes were clinically acceptable, except three discrete kinematic angles (pelvis rotation at initial contact, peak knee flexion during stance and ankle plantarflexion at toe-off). In terms of gait analysis, IMU technology seems sufficiently sensitive to determine gait deviations between PLHIV and SNP.

The IMU and OMC systems demonstrated good agreement, small offsets and acceptable-to-excellent ICCs for all TSP, although less so for double support time and parameters expressed as a percentage of the gait cycle. These findings are similar to those from other validation studies investigating various IMU configurations and reference systems [18, 21–23]. In addition, the observed initial contact and toe-off errors of 0.010 s or less are similar to those that have been reported for IMU systems using smaller recording frequencies and different event-detection algorithms [18, 24].

Double support time and those parameters expressed as a percentage of the gait cycle (especially double support percentage) showed the largest relative differences and/or worst ICCs in both participant groups. Similar results have been reported recently in a study validating a three-IMU system relative to an instrumented walkway [25]. Errors for double support time and percentage, and single support percentage, tended to be lower in PLHIV relative to SNP. These differences may stem from gait speed differences and/or true pathology [25], although further research is needed to support such speculations. Other IMU validation studies comparing (slower walking) older adults to (faster walking) younger adults have had similar findings for these parameters (lower errors for these outcomes in the older adults) [25, 26].

Differences between the IMU and OMC systems were more apparent when comparing (discrete) kinematic angles at specific time points of the gait cycle, and less so when comparing relative joint/segment ROM. This was true for both participant groups. Considering the different technology sources (IMUs versus cameras) as well as models to measure and calculate kinematic angles, these results are not surprising and generally agree with previous studies comparing IMU and OMC technologies [27, 28]. Low RMSE and excellent correlations have for example been reported for most lower limb joints during gait when either using the same biomechanical model to calculate angles from IMU segment position data and OMC marker clusters (RMSE below 5°), or after removing the offset between models (RMSE below 9°) [27, 29, 30]. When however using independent models without offset correction to calculate IMU and OMC kinematics, correlations remained good to excellent while worse RMSE (e.g., up to 28° for the hip) were demonstrated in these studies [27, 30]. Our results reaffirm previous observations that discrete angles are not directly comparable between IMU and OMC systems/models, while relative angular ROM seem more comparable [27]. These observations may largely stem from the different ways that segment positions and joint axes definitions are established during the systems’ respective calibrations—this would be especially true for the sagittal plane. In addition to between-system differences in segment positions and joint axes definitions, soft tissue artifact may affect marker and IMU positions in different ways; further increasing differences between the systems/models [31].

In both participant groups, TSP demonstrated acceptable-to-excellent reliability (except for three parameters), with measurement errors smaller than what would be considered clinically meaningful. For example, the SEM for gait speed—an outcome commonly measured in clinical function studies in PLHIV [9] —was 0.02 m/s (SEM% < 2%) in both groups, whereas a much larger value of 0.1 m/s has been suggested as being clinically significant [32]. In terms of measurement error for TSP, less reliable results were observed for stance time in SNP and single support percentage in both groups. Potential reasons may be that these outcomes were truly unstable, or an insensitivity of the IMU technology to detect a relatively stable phenomenon. In a study by Washabaugh and colleagues [24], where measurement error that is due to natural walking variations was controlled for by means of treadmill walking, stance and swing percentages were the least reliable TSPs measured by foot-mounted IMUs—suggesting larger instrumentation error for these outcomes.

We found that the trends in reliability of IMU-measured kinematic angles were generally similar between PLHIV and SNP groups and fair-to-excellent for all but three angles (pelvis rotation at initial contact in both groups, ankle plantarflexion at toe-off in PLHIV and peak knee flexion in stance in SNP). The worse findings for these discrete outcomes may suggest that some key events of the gait cycle are inherently more variable in the groups, or that the IMU- and OMC-systems were both more susceptible to, and potentially affected in different ways by, soft tissue artifact at these events; considering artifacts reported for the pelvis (high, up to 25 mm), thigh (high: up to 31 mm) and lateral malleolus (moderate: up to 15 mm) [33]. The relative error of these moderate-to-high soft tissue artifacts will be even larger for motions with small ranges (i.e., low signal-to-noise ratios) [23].

For all other joint/segments and planes, ICC values of between 0.76 and 0.99 were observed, with a relatively small SEM (≤ 4.4°). These results are comparable to published results for within-rater or between-trial reliability for OMC [20] and IMU systems [17, 34]. According to a recent systematic review [17], reliability for IMU-measured kinematics across lower limb joints and planes ranged from 0.40 to 0.95 in terms of correlation coefficients and 0.3°–9.9° in terms of absolute errors. However, the authors noted that the small number of studies for each joint did not allow for strong conclusions (e.g., only one study reporting pelvic angles). Most reports of IMU-based systems have not reported the reliability of discrete joint angles; we expand on the existing body of literature in this regard.

In clinical terms, it may be more important to consider absolute rather than relative reliability when interpreting the results for 3D gait analysis—i.e., whether the measurement error renders the instrument meaningful for clinical use [35]. Although the ICC has been widely recommended to assess reliability, it has the disadvantage of being affected by between-participant variability (unlike the SEM). In situations where little variation exists between participants, the ICC will inevitably be low or unmeasurable (as was the case for pelvic obliquity in this study, when calculating an ICC for comparing IMUs and OMC), since it measures the ratio of within-participant variability to between-participant variability [36]. A further example of this limitation is peak knee flexion during stance in SNP, which had a poor ICC of 0.45 but a clinically acceptable SEM of 3.9°. Similarly, the low ICC of pelvis rotation at initial contact was also associated with an acceptable SEM (3.7°) in PLHIV.

When interpreting IMU and OMC data separately to compare gait patterns between PLHIV and SNP, the magnitude and direction of between-group differences (both significant and non-significant) were similar for the two systems. Slowed gait speed—the most consistently reported finding from clinical gait studies in PLHIV [9]—was demonstrated by both systems in terms of clinical but not statistical significance [between-group differences of > 0.10 m/s demonstrate by both systems—exceeding the minimum clinically important difference (MCID) reported for usual-paced gait [32]—but *p*-values for both systems exceeded 0.05]. Both systems also detected significantly increased stance- and double support times in PLHIV (*p* < 0.05), as well as clinically significantly decreased ankle joint angles in PLHIV (between-group differences exceeding 5°). From a gait analysis point of view, these results support the sensitivity of IMUs to the differences between populations on the level of what an HIV-associated deviation might be. As expected, the kinematic differences between PLHIV and SNP in this relatively young sample were mostly small—although the magnitude of biomechanical differences that would translate into functional limitations in PLHIV remains unknown and an area for future research.

### Clinical implications

The validity and reliability of IMU-based gait analyses are not compromised by the presence of HIV. Firstly, this study showed similar trends in validity and reliability in both participant groups. Absolute reliability results indicate a sufficiently low level of measurement error for IMUs to be used for clinical interpretation, and values fall well within the precision error reported for OMC [20]. Secondly, despite the differences in data sources and modeling employed by OMC and IMUs, our results suggest that similar clinical conclusions may be drawn when using either system for clinical gait analysis in PLHIV (e.g., both systems demonstrated the expected kinematic and TSP changes that would logically accompany slow gait in PLHIV relative to SNP). It was not the aim of this paper to describe the deviations potentially occurring in PLHIV, but rather to explore whether an IMU system would be sensitive enough to detect small effects between groups in a similar manner to OMC; indeed this seems to be the case. It should be kept in mind, however, that different IMU systems use different algorithms and because of an inherent offset between IMU- and OMC systems/models, data from IMU and OMC systems should not be used interchangeably.

### Limitations

A study limitation is that only usual-paced walking was assessed, and thus study results are not generalizable to very slow or fast speeds. The accuracy of IMUs are reportedly the highest in the range of 1.0–2.2 m/s, and lower at velocities that are either slower or faster than this range [37]. The performance of IMUs when conducting gait analysis in PLHIV should be verified in such speed ranges. Although IMUs proved to be suited to the specific population used in this project, these results may not readily be assumed to hold true in different populations with gross gait pathology or higher BMI. Although OMC served as reference standard, it is susceptible to faulty marker placement [38]; nevertheless, marker-placement by a laboratory-trained physiotherapist likely limited marker placement errors.

## Conclusion

To conclude, this study demonstrated valid and reliable results from an IMU system for 3D gait analysis, delivering a wide range of clinically relevant gait outcomes in people with and without HIV. Despite the different data sources and modeling used by IMU and OMC systems, a gait analysis conducted in a unique population, namely PLHIV, provided similar magnitudes and directions of differences relative to healthy individuals, and thus similar clinical conclusions are likely when using either system.

## Methods

### Participants

Eight PLHIV and eight SNP were recruited from a public primary care community health center (Table 7). Eligibility criteria included: (1) age 18 to 65; (2) BMI < 25 kg/m^2^; (3) independent ambulatory function; and (4) ability to consent and participate in study procedures. Exclusion criteria were: (1) pregnancy; (2) acute opportunistic infection or illness; (3) peripheral neuropathy; (4) history of major neurological conditions; (5) neuromusculoskeletal impairments or injury affecting usual gait; (6) visual impairment; or (7) acute alcohol consumption. Participants had to have a confirmed laboratory test result of HIV status. The research was in accordance with the Helsinki Declaration and ethical approval was granted by the Stellenbosch University Human Research Ethics Committee (N15/05/043). Written informed consent (including HIV testing consent) was obtained from participants. Pre- and post-test counselling was offered where needed.

**Table 7:** Sample description

Characteristic	SNP (*n* = 8)	PLHIV (*n* = 8)	*p*-value
Age in years, mean (SD)	28.84 (8.11)	36.61 (9.44)	0.10
Female sex, *n* (%)	6 (75)	4 (50)	0.30
Height (m), mean (SD)	1.59 (0.09)	1.64 (0.08)	0.20
Weight (kg), mean (SD)	49.51 (11.24)	57.23 (9.99)	0.17
BMI (kg/m^2^), mean (SD)	19.54 (3.03)	21.19 (3.50)	0.33
Leg length (cm), mean (SD)	84.72 (5.52)	85.86 (4.40)	0.65
Most recent CD4+ T-cell count (cells/µL), mean (SD)	–	558.25 (181.80)	–
Detectable viral load (> 50 cp/mL), *n* (%)	–	6 (75)	–
Years since HIV diagnosis, *n* (%)	–		
< 2 years	–	4 (50)	–
2–5 years	–	2 (25)	–
5–15 years	–	1 (12.5)	–
> 15 years	–	1 (12.5)	–
On ART, *n* (%)	–	6 (75)	–
ART duration in weeks, median (IQR)	–	71.50 (16.00–465.00)	–

*BMI* body mass index, *ART* antiretroviral therapy, *PLHIV* people living with HIV-1 infection, *SNP* HIV-seronegative participants

### Study design

This study incorporated concurrent validity testing and within-session, between-trial repeated measures reliability testing. Concurrent validity refers to a form of criterion validity where the performance of two different measures is assessed at the same time to determine the similarity between the index test/new measure (IMUs in this case) and the criterion measure/reference standard (OMC in this case). Reliability refers to the extent to which repeated measures provide similar results in unchanging individuals [39] and was determined in this study across multiple repeated trials, which all occurred during a single session. A single testing session was thus conducted per participant and a single rater (motion analysis-trained physiotherapist) performed all testing. The study formed part of a larger protocol to study gait features in PLHIV residing in a semi-rural South African setting.

### Setting

Data were collected in the Stellenbosch University Central Analytical Facilities (CAF) 3D Human Biomechanics Unit, Tygerberg Medical Campus, Cape Town, South Africa. Participants were transported between the clinic and the motion laboratory using official university transport services.

### Sample size

Sample size was based on the SEM (a measure of intra-individual variability) for lower limb kinematic angles across the gait cycle, considering a reported SEM of 4.1° [40]. This was the maximum SEM (hip rotation) reported across tri-planar lower limb angular ROM in healthy adults for usual-paced walking [40]. An MCID of 5° is suggested for lower limb gait kinematics [41]. To establish that a measured SEM of 4.1° is lower than 5° at a one-sided 95% confidence interval (CI), the recommendations by Stratford and Goldsmith [42] were followed. The variance ratio $\left( {\frac{{\sigma^{2} }}{{s^{2} }}} \right)$ was calculated as $\left( {\frac{{5.0^{2} }}{{4.1^{2} }}} \right) = 1.5$. Using this variance ratio and Table 7 in [42], the required sample size was estimated for a protocol making use of 6 measurements per participant; i.e., a sample size of 9 participants. Sample size was restricted by pragmatic constraints such as participant transportation; thus, a convenience sample of 8 PLHIV and 8 SNP was deemed practical.

### Instrumentation and procedures

**Fig. 4:**
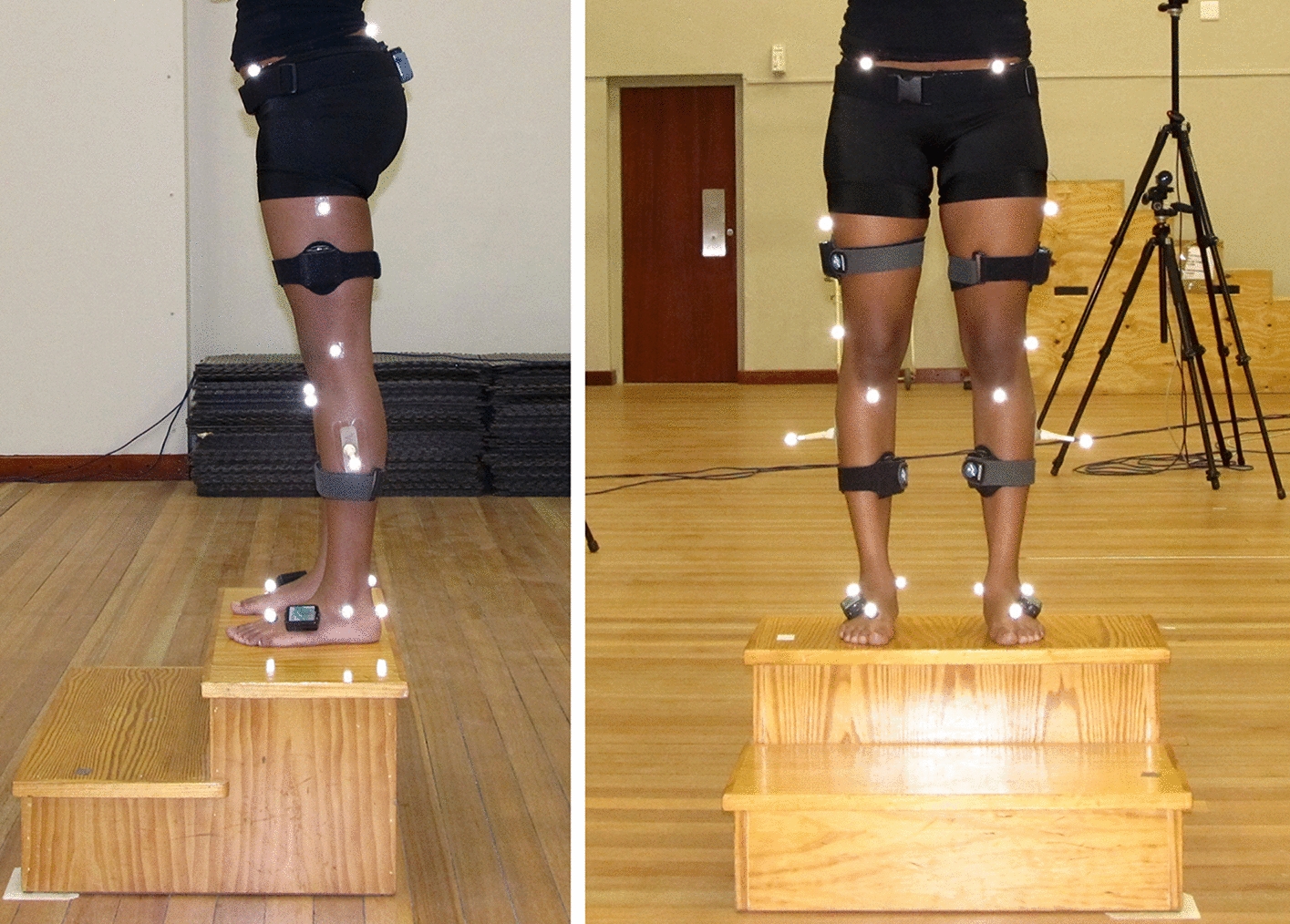
Marker and inertial measurement unit (IMU) placement

IMUs and reflective OMC markers of two independent gait analysis systems were fixated simultaneously on the participant (Fig. 4) to collect gait data concurrently using the two systems and their respective biomechanical models.

#### Inertial measurement unit (IMU) system

The index test was a wireless IMU system (myoMOTION Research Pro, Noraxon USA Inc.) consisting of a receiver and 7 IMUs (for a lower body setup). Each IMU (37.6 mm × 52.0 mm × 18.1 mm; 34 g) has a local coordinate system and measures accelerations and yaw-pitch-roll orientations along three coordinate axes. IMUs were placed on body segments according to a rigid lower body model provided by the IMU system software [myoRESEARCH 3.10.64 (MR3)]. The model considers each body segment as a rigid unit with interlinking joints and assumes a rigid IMU-segment attachment. The system was calibrated before conducting measurements using a neutral standing pose. Gait events (initial contact and toe-off) were detected using an IMU-based contact detection algorithm provided by the software. The algorithm utilizes gyroscope (foot angular velocity) as well as acceleration measurements from the foot-mounted IMU to identify periods when the foot is in contact with the ground, creating virtual foot contact signals for each foot. A sampling rate of 200 Hz was selected for all IMUs. Our laboratory previously demonstrated the capability of the IMUs to measure angles with a static accuracy of 0.4° ± 0.2° (inclination) and 0.8° ± 0.4° (heading) and dynamic accuracy of 0.9° ± 0.2° (inclination) and 2.0° ± 0.8° (heading). Acquired motion-related signals (IMU data) were transmitted wirelessly by a small radio module to a recording laptop.

#### Optical motion capture (OMC) system

The reference standard was an OMC system (MX T-series, VICON Motion Systems Limited) and the Plug-in-Gait (PiG) model. The system uses multiple synchronized high-resolution, high-speed cameras to reconstruct body posture and provides body segment position (origin) and orientation (axis directions) relative to a global fixed coordinate system. The VICON has previously demonstrated high validity and reliability [43], and the Conventional Gait Model (implemented as PiG) constitutes the most widely used and validated biomechanical model in clinical research [44]. This study used 8 infrared tripod VICON T-20 cameras with Nexus 1.8.5 software. The system captured data at 200 Hz. Twenty-two passive retro-reflective markers (14 mm diameter) were placed on anatomical landmarks and biomechanical outcomes were calculated according to a validated modified lower body PiG model provided by the OMC software. Gait events were detected using a time-synchronized, floor-embedded force plate system (Model FP9060-15, Bertec Corporation, Ohio, USA). The OMC capture volume was calibrated prior to data collection.

#### Participant preparation and biomechanical model calibration

Anthropometric measurements were taken as required for the respective systems’ models (height and weight for both systems; leg length from anterior superior iliac spine (ASIS) to medial malleolus and knee- and ankle width for OMC). Markers and IMUs were then placed on the participant simultaneously (Fig. 4). First, 22 OMC markers were placed on bony landmarks according to the PiG model, i.e., bilaterally on the heel (calcaneus at the same height above the plantar foot surface as the toe marker), medial and lateral malleolus, second metatarsal (mid-foot side of equinus break), shank (aligned with the ankle flexion axis), tibial tuberosity, medial and lateral knee (flexion/extension axis), lateral thigh (lower lateral one-third surface), anterior superior iliac spine and posterior superior iliac spine. Markers were not removed during any trials. IMUs were subsequently placed on the sacrum and bilaterally on the lateral thigh (lower segmental quadrant, i.e., the area of lowest muscle belly displacement during walking), shank (anterior and slightly medial to be placed along the tibia), and foot (dorsally and sufficiently proximal to the equinus break to avoid excessive IMU motion) using double-sided tape and Velcro straps. IMU foot-placements were reinforced with elastic adhesive bandage. Participants performed practice trials to familiarize themselves with testing procedures. During practice trials, starting positions for optimal force plate foot strikes were noted, and enough trials were allowed for the rater to be satisfied that a relaxed, normal gait was assumed.

Static anatomical OMC calibration was subsequently performed with the participant standing on the force plate according to standard laboratory protocol (once off per participant and prior to IMU calibration). Next, the IMU model was calibrated as per the manufacturer’s instructions by having the participant stand stationary in a neutral reference posture. The calibration was performed on a 30 cm-high wooden platform to mitigate potential floor-based magnetic distortions. As frequent IMU calibration within test series is recommended [45] to avoid drift over time, the IMU system was calibrated repeatedly (directly prior to each gait trial). We previously demonstrated within-rater reliability for this calibration pose and procedure (SEM 0.3°–2.2°; Berner, K., Cockcroft, J., Morris, L., & Louw, Q., under review).

#### Gait analysis protocol

Each session consisted of a neutral-pose IMU calibration, directly followed by a barefooted walking trial during which IMU and OMC data were recorded simultaneously. Participants had to walk at a self-selected usual speed along a straight 10-m walkway with the force plate system embedded midway. Participants started walking approximately 1 m before a taped line on the floor and ended after crossing a second line. Time synchronization between the two systems was performed automatically using alignment of a hardwire synchronization pulse (5 V TTL signal). A gait trial was deemed successful if the participant’s entire landing foot contacted at least one force plate without obvious targeting. Gait trials were performed in the same direction each time and after each trial, the participant returned to the wooden platform immediately for the next IMU calibration. Trials continued until good-quality data for 6 trials (3 left- and 3 right-footed force plate strikes) were obtained.

### Data processing

The IMU system software automatically filtered raw data using a robust fusion algorithm (Kalman filter) optimized for IMU data. Angular orientations were estimated at IMU level by combining the elemental sensor component axes readings into four element quaternion values. Segment dimensions of the model were calculated in the IMU software using participant height to estimate anthropometric dimensions. For the determination of distance-related outcomes, the following applied: the IMU software estimated model translation over the ground using a forward kinematics technique together with sequential pinning of the foot segments onto the ground during the contact phase. The bone segment lengths on the biomechanical model were scaled to the participant dimensions. Then, using their measured orientations and known lengths, the interconnected lower limb segments were positioned in space in a kinematic chain, which translates the model forwards from foot contact to subsequent foot contact. Outcomes such as step length were then extracted from the position of virtual landmarks on the foot segments.

Pre-processing of OMC gait trials was done in Nexus software. Marker trajectories were reconstructed and labeled using standard functions, then smoothed with a fourth-order, zero-lag low-pass Butterworth filter (6 Hz cut-off) [46]. Joint and segment kinematics were calculated using the standard dynamic PiG pipeline, which determines hip joint centers using the Davis equations [47]. Knee axis estimation was performed by optimizing the thigh-rotation offset parameter during gait [48], and ankle axis estimation by determining the shank-rotation offset parameter during the static trial using medial and lateral malleolus markers. OMC gait events were detected from force plate data (20 N threshold).

Data recorded in the IMU and OMC software were, respectively, exported to single .csv and .c3d files and imported into MATLAB software (R2017a, MathWorks). In cases where a gait trial contained more than one complete and valid gait cycle for one or both legs, only the gait cycle for each leg judged to contain the best data quality was retained for analysis. The cyclical gait events from each system (initial contact and toe-off) were used to segment trial data into cycles normalized in time to 101 data points at 1%-time intervals. Gaps in gait trajectories were inspected and filled and IMU and OMC outcomes were determined using custom analysis scripts, including the construction of visualizations. Although the IMU biomechanical model is based on the standards for joint rotations sequences as set by the International Society of Biomechanics (ISB), some differences in conventions exist regarding polarity. Thus, opposing angular polarities in IMU output were manually inverted according to the positive definitions of each angular motion before determining IMU outcomes. Finally, using a macro routine, all outcomes were exported to MS Excel for further analysis.

### Data outcomes and analysis

Using the average of both lower limbs, TSP (temporal, spatial, temporophasic and temporospatial parameters) and kinematic angular outcomes were selected based on clinical relevance in discriminating elderly and/or fall-prone gait [49]. Kinematics included ROM (difference between the maximum and minimum angle during gait cycle) and key point values (angular values at specific time points of gait cycle). Performance of the IMU system’s event-detection algorithm was evaluated by calculating the average detection offset [difference in milliseconds (ms) between IMU- and OMC-detected events] for initial contact and toe-off. Detection offsets were averaged across all participants and converted from frames per second to seconds using the sampling rate of the system, and then converting the result to ms. Extraction of kinematic key points from the time-normalized average of each assessment was performed using a customized routine in MATLAB, based on key event and phase definitions listed in Additional file 1.

### Statistical analysis

Statistical analysis was performed using MS Excel V16.12 (Bland–Altman and LoA) and IBM SPSS Statistics 25.0 (SPSS Inc., Chicago, IL, USA) (all other analyses). Between-group differences were assessed using independent t-tests (separately for each system). Statistical significance was set at *p* < 0.05. An MCID of > 5° [20] was considered clinically significant for kinematic angles, while clinically significant differences for TSP were interpreted in terms of percentage differences and reported MCIDs from the literature, where available.

#### Concurrent validity

Concurrent validity between the mean gait outcomes measured by the two systems was assessed by calculating ICCs (two-way mixed, mean rating) with 95% CIs. Bland–Altman bias (indicative of magnitude-dependent systematic difference) and 95% LoA (representing limits around the mean difference within which 95% of observed differences lie) were calculated. Absolute offset error between IMU- and OMC-measured outcomes was assessed using RMSE. To aid clinical interpretation of TSP, mean absolute percentage errors were calculated.

#### Within-session reliability

Relative within-session reliability of IMU-measured gait outcomes was assessed by using all six trials for each outcome and calculating ICCs (two-way mixed effects, single rater/measurement) with 95% CIs. Absolute reliability was quantified using SEM, calculated as the square root of the mean-square-error (MS_E_) from a two-way repeated-measure analysis of variance (ANOVA). This approach is robust to between-participant variability and thus preferable to SEM calculations based on the ICC [19]. The upper 95% CL of the SEM was calculated according to [19] by multiplying the SEM by a multiplying factor determined according to the number of participants and trials (1.2 in this study). For TSP, absolute %SEM was additionally calculated to aid interpretation.

The ICC is an index ranging from 0 to 1 that reflects both degree of correlation and agreement between measurements within a class of data. ICCs were interpreted as poor (ICC < 0.60), questionable (0.60 ≤ ICC < 0.70), acceptable (0.70 ≤ ICC < 0.80), good (0.80 ≤ ICC < 0.90) or excellent (ICC ≥ 0.90) [50]. Absolute agreement criteria were used for all ICCs. To interpret mean absolute percentage error and %SEM, a classification criterion of acceptability was used based on standard statistical thresholds for significance analysis [51]. It was also considered that a reference threshold of 5% has been proposed for accuracy error in step length and distance [51]. The following categories were considered: excellent (< 5%), good (5% to < 10%), sufficient (10% to < 20%) and unacceptable (20% or higher) [51].

## Supplementary information

**Additional file 1.** Delamination and definition of gait phases, including defining events. Key event and phase definitions used in the customized MATLAB routine to extract kinematic key points and phases from the time-normalized average of each assessment.

## Data Availability

The datasets used and/or analyzed during the current study are available from the corresponding author on reasonable request.

## Abbreviations

ART: Antiretroviral therapy
BMI: Body mass index
CL: Confidence limits of standard error of measurement
HIV: Human immunodeficiency virus
IMU: Inertial measurement unit
LoA: Limits of agreement
MCID: Minimum clinically important difference
OMC: Optical motion capture
PiG: Plug-in-Gait
PLHIV: People living with HIV
RMSE: Root-mean-square error
ROM: Range of motion
SEM: Standard error of measurement
SNP: HIV-seronegative participants
TSP: Time–space gait parameters/temporal, spatial, temporophasic and temporospatial parameters

## References

[1] Price RW, Brew B, Sidtis J, Rosenblum M, Scheck AC, Cleary P (1988). The brain in AIDS: central nervous system HIV-1 infection and aids dementia complex. Science.

[2] Richert L, Dehail P, Mercié P, Dauchy F, Bruyand M, Greib C (2011). High frequency of poor locomotor performance in HIV-infected patients. AIDS..

[3] Scott WB, Oursler KK, Katzel LI, Ryan AS, Russ DW (2007). Central activation, muscle performance, and physical function in men infected with human immunodeficiency virus. Muscle Nerve.

[4] Ellis RJ, Rosario D, Clifford DB, McArthur JC, Simpson D, Alexander T (2010). Continued high prevalence and adverse clinical impact of human immunodeficiency virus-associated sensory neuropathy in the era of combination antiretroviral therapy. Arch Neurol.

[5] Sullivan E, Rosenbloom M, Rohlfing T, Kemper C, Deresinski S, Pfefferbaum A (2011). Pontocerebellar contribution to postural instability and psychomotor slowing in HIV infection without dementia. Brain imaging Behav..

[6] Greene M, Covinsky KE, Valcour V, Miao Y, Madamba J, Lampiris H (2015). Geriatric syndromes in older HIV-infected adults. JAIDS J Acquir Immune Defic Syndr..

[7] Erlandson KKM, Allshouse AAAA, Jankowski CCM, Duong S, MaWhinney S, Kohrt WWM (2012). Risk factors for falls in HIV-infected persons. J Acquir Immune Defic Syndr.

[8] Berner K, Strijdom H, Essop MF, Webster I, Morris L, Louw Q (2019). Fall history and associated factors among adults living with HIV-1 in the Cape Winelands, South Africa: an exploratory investigation. Open Forum Infect Dis..

[9] Berner K, Morris L, Baumeister J, Louw Q (2017). Objective impairments of gait and balance in adults living with HIV-1 infection: a systematic review and meta-analysis of observational studies. BMC Musculoskelet Disord..

[10] Greene M, Justice AC, Covinsky KE (2017). Assessment of geriatric syndromes and physical function in people living with HIV. Virulence..

[11] Chen S, Lach J, Lo B, Yang G-Z (2016). Toward pervasive gait analysis with wearable sensors: a systematic review. IEEE J Biomed Heal Informatics..

[12] Iosa M, Picerno P, Paolucci S, Morone G (2016). Wearable inertial sensors for human movement analysis. Expert Rev Med Devices.

[13] Picerno P (2017). 25 years of lower limb joint kinematics by using inertial and magnetic sensors: a review of methodological approaches. Gait Posture..

[14] Kavanagh JJ, Menz HB (2008). Accelerometry: a technique for quantifying movement patterns during walking. Gait Posture..

[15] Mo S, Chow DHK (2018). Accuracy of three methods in gait event detection during overground running. Gait Posture..

[16] Lindemann U, Zijlstra W, Aminian K, Chastin SFM, de Bruin ED, Helbostad JL (2014). Recommendations for standardizing validation procedures assessing physical activity of older persons by monitoring body postures and movements. Sensors..

[17] Poitras I, Dupuis F, Bielmann M, Campeau-Lecours A, Mercier C, Bouyer LJ (2019). Validity and reliability of wearable sensors for joint angle estimation: a systematic review. Sensors..

[18] Teufl W, Lorenz M, Miezal M, Taetz B, Fröhlich M, Bleser G (2018). Towards inertial sensor based mobile gait analysis: event-detection and spatio-temporal parameters. Sensors..

[19] Hopkins WG (2000). Measures of reliability in sports medicine and science. Sports Med.

[20] McGinley JL, Baker R, Wolfe R, Morris ME (2009). The reliability of three-dimensional kinematic gait measurements: a systematic review. Gait Posture..

[21] Kluge F, Gaßner H, Hannink J, Pasluosta C, Klucken J, Eskofier B (2017). Towards mobile gait analysis: concurrent validity and test-retest reliability of an inertial measurement system for the assessment of spatio-temporal gait parameters. Sensors..

[22] Donath L, Faude O, Lichtenstein E, Nüesch C, Mündermann A (2016). Validity and reliability of a portable gait analysis system for measuring spatiotemporal gait characteristics: comparison to an instrumented treadmill. J Neuroeng Rehabil..

[23] Bertoli M, Cereatti A, Trojaniello D, Avanzino L, Pelosin E, Del Din S (2018). Estimation of spatio-temporal parameters of gait from magneto-inertial measurement units: multicenter validation among Parkinson, mildly cognitively impaired and healthy older adults. Biomed Eng Online..

[24] Washabaugh EPE, Kalyanaraman T, Adamczyk PPG, Claflin ESEES, Krishnan C (2017). Validity and repeatability of inertial measurement units for measuring gait parameters. Gait Posture..

[25] Morris R, Stuart S, McBarron G, Fino PC, Mancini M, Curtze C (2019). Validity of MobilityLab (version 2) for gait assessment in young adults, older adults and Parkinson’s disease. Physiol Meas.

[26] Schmitz-Hübsch T, Brandt AU, Pfueller C, Zange L, Seidel A, Kühn AA (2016). Accuracy and repeatability of two methods of gait analysis—GaitRiteTM und Mobility LabTM—in subjects with cerebellar ataxia. Gait Posture..

[27] Nüesch C, Roos E, Pagenstert G, Mündermann A (2017). Measuring joint kinematics of treadmill walking and running: comparison between an inertial sensor based system and a camera-based system. J Biomech.

[28] Al-Amri M, Nicholas K, Button K, Sparkes V, Sheeran L, Davies J (2018). Inertial measurement units for clinical movement analysis: reliability and concurrent validity. Sensors..

[29] Picerno P, Cereatti A, Cappozzo A (2008). Joint kinematics estimate using wearable inertial and magnetic sensing modules. Gait Posture..

[30] Ferrari A, Cutti AG, Garofalo P, Raggi M, Heijboer M, Cappello A (2010). First in vivo assessment of “Outwalk”: a novel protocol for clinical gait analysis based on inertial and magnetic sensors. Med Biol Eng Comput..

[31] Zügner R, Tranberg R, Timperley J, Hodgins D, Mohaddes M, Kärrholm J (2019). Validation of inertial measurement units with optical tracking system in patients operated with Total hip arthroplasty. BMC Musculoskelet Disord..

[32] Bohannon RW, Glenney SS (2014). Minimal clinically important difference for change in comfortable gait speed of adults with pathology: a systematic review. J Eval Clin Pract..

[33] Peters A, Galna B, Sangeux M, Morris M, Baker R (2010). Quantification of soft tissue artifact in lower limb human motion analysis: a systematic review. Gait Posture..

[34] Cho YS, Jang SH, Cho JS, Kim MJ, Lee HD, Lee SY (2018). Evaluation of validity and reliability of inertial measurement unit-based gait analysis systems. Ann Rehabil Med..

[35] Bruton A, Conway JH, Holgate ST (2000). Reliability: what is it, and how is it measured?. Physiotherapy..

[36] Koo TK, Li MY (2016). A guideline of selecting and reporting intraclass correlation coefficients for reliability research. J Chiropr Med..

[37] Lützner C, Voigt H, Roeder I, Kirschner S, Lützner J (2014). Placement makes a difference: accuracy of an accelerometer in measuring step number and stair climbing. Gait Posture..

[38] Della Croce U, Leardini A, Chiari L, Cappozzo A, Della Croce U, Leardini A (2005). Human movement analysis using stereophotogrammetry. Gait Posture..

[39] de Vet HCW, Terwee CB, Knol DL, Bouter LM (2006). When to use agreement versus reliability measures. J Clin Epidemiol.

[40] Meldrum D, Shouldice C, Conroy R, Jones K, Forward M (2014). Test–retest reliability of three dimensional gait analysis: including a novel approach to visualising agreement of gait cycle waveforms with Bland and Altman plots. Gait Posture..

[41] Wilken JM, Rodriguez KM, Brawner M, Darter BJ (2012). Reliability and minimal detectible change values for gait kinematics and kinetics in healthy adults. Gait Posture..

[42] Stratford PW, Goldsmith CH (1997). Use of the standard error as a reliability index of interest: an applied example using elbow flexor strength data. Phys Ther..

[43] Ehara Y, Fujimoto H, Miyazaki S, Mochimaru M, Tanaka S, Yamamoto S (1997). Comparison of the performance of 3D camera systems II. Gait Posture..

[44] Baker R, Leboeuf F, Reay J, Sangeux M, Sangeux M (2017). The conventional gait model—success and limitations. Handbook of human motion.

[45] Noraxon. myoMOTION sensor and receiver user manual; 2018. https://www.noraxon.com/noraxon-download/myomotion-system-user-manual/. Accessed 7 Mar 2019.

[46] Yu B, Gabriel D, Noble L, An K-N (1999). Estimate of the optimum cutoff frequency for the butterworth low-pass digital filter. J Appl Biomech..

[47] Davis RB, Õunpuu S, Tyburski D, Gage JR (1991). A gait analysis data collection and reduction technique. Hum Mov Sci.

[48] Schache AG, Baker R, Lamoreux LW (2006). Defining the knee joint flexion–extension axis for purposes of quantitative gait analysis: an evaluation of methods. Gait Posture..

[49] Berner K. Biomechanical analysis of specific motor impairments contributing to early functional decline in adults living with HIV-1 infection: a sub-study to the Cape Winelands HAART to HEART (Prevalence)/EndoAfrica study. SUNScholar; 2019. http://scholar.sun.ac.za/handle/10019.1/105865. Accessed 23 May 2019.

[50] Post MW (2016). What to do with “moderate” reliability and validity coefficients?. Arch Phys Med Rehabil.

[51] Fusca M, Negrini F, Perego P, Magoni L, Molteni F, Andreoni G (2018). Validation of a wearable IMU system for gait analysis: protocol and application to a new system. Appl Sci..

