# Supplementary material for: Kinematics and temporospatial parameters during gait from inertial motion capture in adults with and without HIV: a validity and reliability study
Source: Biomed Eng Online. 2020 Jul 24;19:57. doi: 10.1186/s12938-020-00802-2 (PMC7379351; doi:10.1186/s12938-020-00802-2)
Supplement: Supplementary file 1 — Additional file 1. Delamination and definition of gait phases, including defining events. Key event and phase definitions used in the customized MATLAB routine to extract kinematic key points and phases from the time-normalized average of each assessment. [file 12938_2020_802_MOESM1_ESM.docx]

**Additional file 1.**

**Table A. Delamination and definition of gait phases, including defining events.**

| **Phase or sub phase** | **Definition** | **Defining events** |
| --- | --- | --- |
| Gait cycle (stride) | Constitutes the basic unit of gait and is defined as the time period between two successive occurrences of one of the repetitive events during walking; by convention foot contact with the ground | IC1 to IC2 |
| Stance phase | | IC1 to TO |
| Loading response/first double support | Period of weight acceptance, starting from initial contact to opposite toe-off. Corresponds to first double support phase. | IC1 to OTO |
| Mid stance | The first half of single support, lasting from opposite toe-off to heel rise of ipsilateral foot. | OTO to HR |
| Terminal stance | The second half of single support, lasting from ipsilateral heel rise to opposite initial contact. | HR to OIC |
| Pre-swing/second double support | The second double support phase, lasting from opposite initial contact to ipsilateral toe-off. | OIC to TO |
| Swing phase | | TO to IC2 |
| Initial swing | Period from toe-off to instant when swing leg is adjacent to stance limb. | TO to FA |
| Loading response and mid stance periods |  | LR (begin) to MSt (end) |
| Mid stance and terminal stance periods |  | MSt (begin) to TSt (end) |
| A1 | Portion of the GC corresponding to the A1 power phase of the ankle: a region of negative power, corresponding to eccentric plantar flexor activity at the ankle during midstance and terminal stance | Maximum ankle plantarflexion in MSt to maximum ankle dorsiflexion in TSt |
| A2 | Portion of the GC corresponding to the A2 power phase of the ankle: a region of positive power, corresponding to the concentric burst of propulsive plantar flexor activity during pre-swing. | HR to TO |
| K1 | Portion of the GC corresponding to the K1 power phase of the knee: a region of negative power, corresponding to eccentric knee extensor activity at during loading response. | IC1 to maximum knee flexion in stance |
| K2 | Portion of the GC corresponding to the K2 power phase of the knee: a region of positive power, corresponding to concentric knee extensor activity during midstance. This is followed by a period of negligible joint power during that period of time when the ground reaction force stabilizes the knee in extension. | Maximum knee flexion in MSt to maximum knee extension in TSt |
| K3 | Portion of the GC corresponding to the K3 power phase of the knee: a region of negative power, corresponding to eccentric activity in the rectus femoris during pre-swing. At normal or slightly faster walking speeds, rectus femoris controls knee flexion. | Maximum knee extension in TSt to maximum knee flexion in swing |
| H3 | Portion of the GC corresponding to the H3 power phase of the hip: a region of positive power, corresponding to concentric activity in the hip flexors during pre-swing and initial swing. Sometimes called "pull off", this is the muscular system's second largest contribution of propulsive power during the gait cycle. | Maximum hip extension in stance to maximum hip extension in swing |
| Abbreviations: GC = gait cycle; H3 = H3 power phase of hip; HR = heel rise; IC = initial contact; K3 = K3 power phase of knee; LR = loading response; MSt = mid stance; ROM = range of motion; TO = toe-off; TS = terminal stance. | | |
